# Gene therapy for hearing loss: challenges and the promise of cellular plasticity and epigenetic modulation

**DOI:** 10.3389/fneur.2024.1511938

**Published:** 2024-12-11

**Authors:** Samprita Das, Uri Manor

**Affiliations:** Department of Cell and Developmental Biology, School of Biological Sciences, University of California, San Diego, La Jolla, CA, United States

**Keywords:** hearing loss, gene therapy, partial reprogramming, epigenetics, adeno-associated virus, HDAC inhibitors

## Abstract

Hearing loss can profoundly impact an individual’s quality of life, affecting communication, social interactions, and overall well-being. Many people with hearing impairment report feelings of isolation, frustration, and decreased confidence in social settings, which can lead to withdrawal from activities they once enjoyed. Genetics plays a significant role in congenital hearing loss, accounting for approximately half of all cases. While gene therapy holds immense promise for restoring hearing function in cases of hereditary hearing loss (HHL), current methods face certain challenges that must be overcome to successfully develop therapeutic approaches. This review will explore these challenges and offer a perspective on how epigenetic modulation has the potential to address them, potentially revolutionizing the treatment of genetic hearing disorders.

## Sensorineural hearing loss: an overview

Most hereditary hearing loss (HHL) is sensorineural (SNHL), resulting from defects in sound processing in the auditory sensory system. SNHL can occur anywhere from the cochlea to the auditory cortex and at any time from early development *in utero* through to middle age and beyond (1).

**Figure 1:**
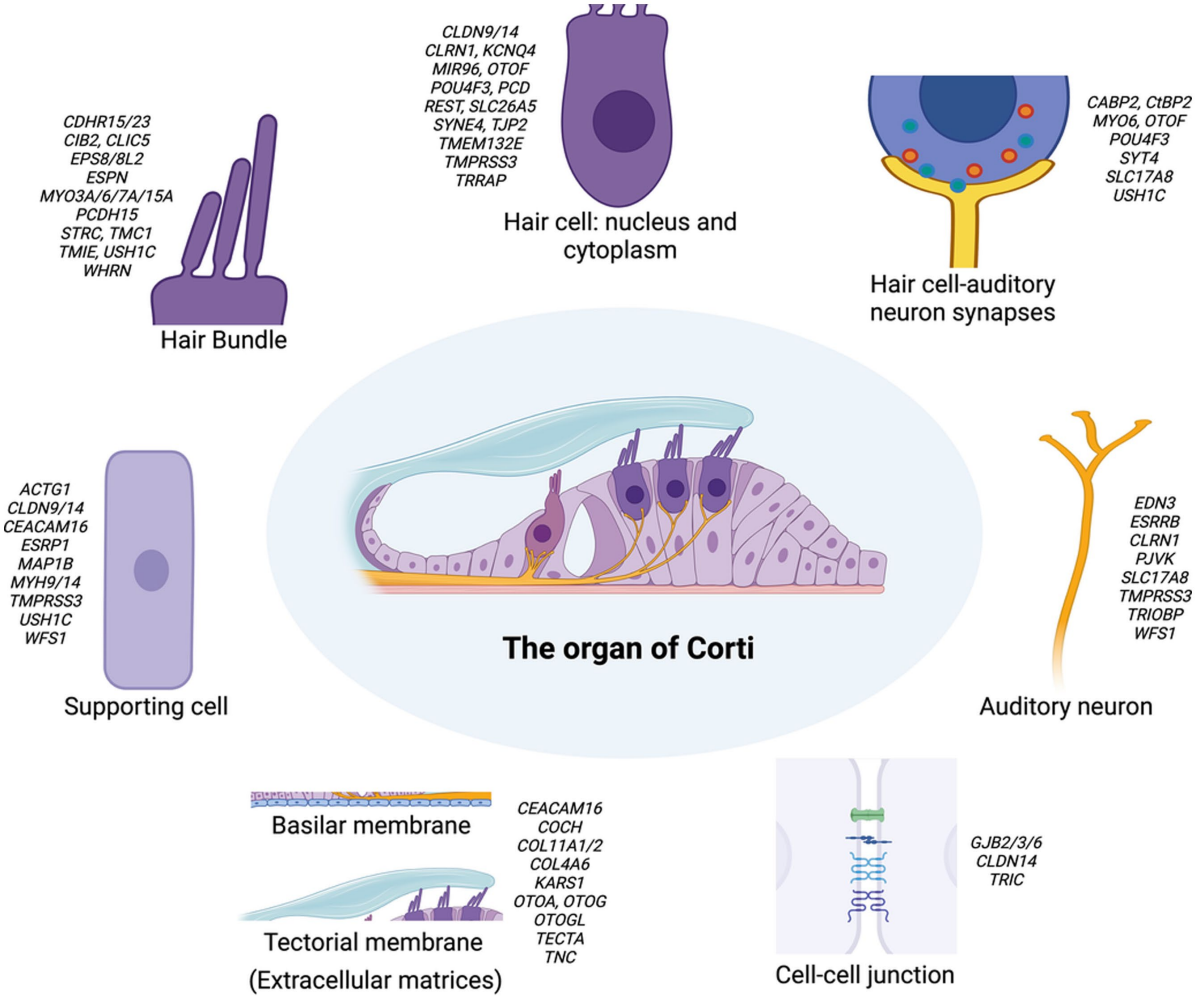
Schematic representation of the organ of Corti and its associated key structures in the inner ear. The central image depicts the overall structure of the organ of Corti. Surrounding this are detailed illustrations of key components: the hair bundle, hair cell (nucleus and cytoplasm), supporting cell, hair cell-auditory neuron synapses, and auditory neuron. The basilar membrane, tectorial membrane, and cell–cell junction are shown separately. Each component is annotated with genes associated with hearing loss. This figure highlights the complex genetic landscape underlying hereditary hearing loss and the diverse cellular structures involved in auditory function. This schematic representation provides a simplified overview of the major structural components without depicting precise anatomical proportions between inner and outer hair cells or their bundles. For clarity and focus on key genetic elements, specialized structures such as inner phalangeal cells, border cells, and the tonotopic organization of the tectorial membrane are not illustrated, though they play important roles in auditory function.

The cochlea is a crucial component of our auditory system, containing intricate microstructures. Multiprotein complexes that form key cochlear structures and act in molecular pathways essential to cochlear functions have been identified as encoded by deafness-related genes. These genes fall into three major categories depending on their expressing cell type: expressed in hair cells, supporting cells, or stria vascularis. The organ of Corti in the cochlea contains two types of hair cells (HCs): inner hair cells (IHCs) and outer hair cells (OHCs). IHCs transduce sound vibrations into electrical signals, while OHCs mainly mechanically amplify sound signals (2–4). Stereocilia, actin-based membrane protrusions on the apical surface hair cells, are essential for mechanotransducing sound vibrations. For proper hearing, the tallest row of stereocilia must form a junction with the tectorial membrane (TM) (5, 6). More than half of deafness-related genes are expressed in HCs, including *MYO15A*, *MYO7A*, *USH1C*, *CDH23*, *MYO6*, *TMC1*, *PCDH15* (important for stereocilia function), *OTOF*, *VGlut3, SRRM4* (confined to IHCs), *STRC*, *KCNQ4*, *INSM1*, *BCL11B*, and *SYNE4* (confined to OHCs) (Figure 1) (7–12).

Supporting cells (SCs) surround HCs, maintain their structural integrity, and generate extracellular factors to promote mechanotransduction and remove excess neurotransmitters or potassium (13, 14). Common causes of deafness in SCs include mutations in genes encoding gap junction proteins, such as *GJB2* (Connexin 26), *GJB6* (Connexin 30), and *GJB3* (Connexin 31) (9, 15) (Figure 1). The stria vascularis (SV) lines the lateral wall of the cochlea and plays a key role in maintaining a high K^+^ concentration in the endolymph, which is crucial for HC function. The SV has three cell layers: marginal cells, intermediate cells, and basal cells, which work together to pump K^+^ through ion channels from the perilymph to the endolymph (16, 17). Common deafness-related genes in the SV include those related to ion channels and their regulatory subunits (*KCNQ1*, *KCNE1* in marginal cells; *KCNJ10* in intermediate cells) or tight junction molecules required for cell layer integrity (*CLDN11*). Recent studies have highlighted pendrin’s critical role in K^+^ transport, demonstrating that pendrin helps maintain endolymphatic K^+^ homeostasis, which is essential for proper cochlear function (18). There is also evidence for mitochondrial NSHL mutations primarily impacting the SV, which is perhaps a reflection of the uniquely high metabolic/energetic demand on the SV and cochlea in general (19).

## Current SNHL therapeutic approaches

Hearing loss is the most common sensory disorder while the most common cause of hearing loss is age-related progressive loss of inner ear sensory hair cells, mostly affected from OHC in the base progresses with age (1). Humans do not naturally regenerate hair cells or spiral ganglion neurons. However, certain mutations can correct for better function if identified early. Gene therapy has emerged as a promising approach for treating hereditary sensorineural hearing loss (SNHL) as it can correct or modify the faulty genes. The choice of therapy depends on the nature of the pathogenic variants: loss-of-function or haploinsufficient variants are addressed through gene replacement or editing, while gain-of-function variants are targeted for deletion, inactivation, or replacement (9). Dominant-negative alleles, on the other hand, are deleted or inactivated to allow the healthy allele to function. Several delivery methods are currently being explored, such as gene replacement by adeno-associated viruses, exosomes, gene editing by CRISPR-Cas9, gene suppression by antisense oligonucleotides and RNA interference, etc. (9, 20).

### Adeno-associated viruses

Known for its low pathogenicity and immunogenicity, AAV is an emerging choice of vector delivery (21, 22). Recent studies have focused on modifying AAV capsids to improve their efficacy. For example, AAV2/Anc80L65 was used to deliver the *Tmc1* gene, resulting in hearing threshold improvements of up to 40 dB and transduction of half the outer hair cells (OHCs) (23). A novel vector, AAV9-PHP.B, achieved 100% OHC transduction when delivering the *Tmc1* gene via the utricle (24, 25). Despite its high safety profile and efficiency in cell specificity, its limited packaging capacity (~4.7 kb) restricts its usage in delivering large therapeutic sequences (26). To overcome this limitation, dual-AAV and triple-AAV strategies can be utilized (27, 28). For instance, dual AAV2/6 was used to deliver split-*Otof* cDNA, improving acoustic function with a 20–30 dB threshold improvement in low frequencies (29). A significant breakthrough came with the dual AAV approach to deliver the Otoferlin gene (AAV1-hOTOF), which successfully restored hearing in four out of five children treated (30).

### Liposomes and exosomes

Offering an advantage over AAVs, non-viral strategies, such as liposomes, lipid nanoparticles (LNPs), and exosomes, can accommodate larger therapeutic molecules (31). Liposomes, while versatile, may have lower transfection rates and potential toxicity due to their unnatural lipid content (32). Exosomes, a more recent development, can be modified for increased functionality and cell-specific targeting (33). Exosome-associated AAVs (exo-AAVs) have shown higher transduction efficiency than conventional AAVs, with exo-AAV1 successfully used to deliver the *Lhfpl5* gene in mouse models, improving ABR threshold at low frequencies (34). While boasting considerable advantages, these methods often exhibit reduced transduction efficiency in comparison to viral approaches.

### CRISPR-Cas9 genome editing

CRISPR-Cas9 genome editing technology allows for precise genetic modifications (35). This system generates DNA double-strand breaks (DSB), which can be repaired through homology-directed repair (HDR) or non-homologous end joining (NHEJ). HDR is a highly accurate repair mechanism that can correct mutations or insert large DNA fragments, but it only works in dividing cells during the S and G2 phases of the cell cycle. NHEJ, while error-prone, functions throughout the cell cycle, including in non-dividing cells. Since cochlear cells in both mice and humans are non-dividing at birth, postnatal gene editing in these cells using CRISPR-Cas9 is limited to NHEJ-mediated loss-of-function edits, which primarily silence targeted alleles. In a notable study, CRISPR-Cas9 editing was used to silence mutant alleles in mice carrying mutations in the *Myo6* and *Kcnq4* genes, demonstrating its potential for treating dominant mutations (36–38). Another CRISPR approach is base-editing, which is potentially more efficient and has shown success for treating hearing loss caused by mutations in stereocilin (39). However, the efficiency of CRISPR-Cas9 editing in cochlear cells remains low, with most studies reporting editing rates of less than 10% of target cells, which significantly limits the therapeutic potential of this approach (38).

### Antisense oligonucleotides

ASOs are synthetic nucleic acids that can modulate gene expression by binding to complementary RNA sequences (40). They can act as gene suppression tools by inhibiting mutant gene expression through degrading target mRNA or modulating mRNA’s alternative splicing. For example, ASO-29 was used to block an aberrant splice site in the *USH1C* gene, restoring auditory and vestibular function in a mouse model when injected early in development (41, 42). ASOs can be administered “naked” or within lipid nanoparticles through various routes, including intravenous injections, intraperitoneal injections, and local injection in the inner ear, muscle, or nervous system. This flexibility in administration and the fact that they do not require viral vectors are significant advantages.

### RNA interference

With the advantage of highly specific targeting ability, RNAi uses small RNA molecules to inhibit gene expression through neutralizing targeted RNA molecules. This approach typically relies on one of two types of small non-coding RNA molecules: short interfering RNAs (siRNAs) and microRNAs (miRNAs) (43). In one study, siRNA was used to silence a dominant negative variant of *GJB2* (R75W) and prevent hearing loss (44). Another study used miRNA targeting *Tmc1*, delivered by AAV2/9, which delayed the progression of hearing loss for up to 35 weeks in neonatal mice (45). However, RNAis may require repeated administration for long-term effect and can have off-target effects, which are potential disadvantages. A related approach that could in theory require fewer repeated administration is expression of short hairpin RNAs via lentivirus (46).

## Challenges in SNHL gene therapy

Gene therapy holds immense promise for restoring hearing in cases of genetic hearing loss. However, there remain some critical roadblocks to overcome to make this promise a reality, ranging from the optimal timing of intervention to ensuring the long-term efficacy of treatments.

### Critical window for gene therapy

The timing of gene therapy intervention is crucial for successful treatment of sensorineural hearing loss (SNHL). According to Minoda et al. (47), three important time points must be considered: the normal gene expression initiation time, the normal gene mature expression time, and the phenotypic manifestation time (47). Ideally, treatment should be administered at or before the normal gene expression initiation time, and at the latest before phenotypic manifestation. This timing is particularly challenging for conditions like Cx26 mutation-induced hearing loss, which manifests at birth, potentially necessitating *in utero* gene delivery (48, 49). Early detection and treatment of SNHL is vital, as early intervention has significant benefits, including better speech and language scores. This has been described as a “Neurodevelopmental emergency” (50, 51). Recognizing this urgency, new guidelines established by the Joint Commission of Infant Hearing in 2019 set the goal of “1-2-3”: screen by 1 month, diagnose by 2 months, and intervene by 3 months of age.

Another significant challenge in translating mouse model studies to human applications is the heterochrony between mouse and human auditory system development (9, 47). While human cochlear maturation is well-advanced at mid-gestation and complete at birth, mice are born deaf and their cochlea reach maturation around postnatal day 20 (P20). Most successful interventions in mouse models have been performed during the early neonatal period (P0–P2), which corresponds to embryonic stages in humans. This timing disparity means that many proof-of-concept studies in mice are assessing prevention rather than restoration of hearing impairment, as interventions performed shortly before hearing onset (P8–P11) have been ineffective.

The focus on prevention in mouse models presents another significant limitation in translating these findings to human applications. For instance, positive effects in mouse models have primarily been reported for interventions during the early neonatal period (P0-P2, occasionally extending to P7). However, given the different developmental timelines, these interventions in mice would correspond to embryonic stages in humans. This discrepancy highlights the need for developing strategies that can address fully matured cochlea, which in mice would require interventions from approximately P30 onwards. To date, genuine restoration of hearing impairment in mature systems have been achieved in only two mouse mutants: Vglut3^−/−^ and Otof^−/−^, both of which involve defects in inner hair cell synaptic vesicle proteins (52, 53). Notably, human clinical trials with dual AAV-mediated delivery of Otoferlin have also shown success, but raise yet another critical window of therapeutic success that cochlear implant research has already anticipated: If hearing is not restored by the age of 5 or 6, it is unlikely that auditory language processing will be possible (30).

### Longevity of therapeutic effects

The durability of gene therapy effects in the inner ear is a significant concern (9). In theory the effects could be long-lasting due to the terminally differentiated nature of many inner ear cell types; practical results have varied greatly from a few weeks to months or a year. This has also varied by the specific genes involved and the animal models used.

Several factors contribute to the reduction in efficacy over time. These include the generation of neutralizing antibodies against viral vectors and exogenous genes, and progressive inner ear cell degeneration when editing efficiency is low. Additionally, the choice of promoters and AAV serotypes plays a crucial role in long-term gene expression (54–56). Different promoters and AAV serotypes are subject to varying levels of epigenetic modifications, such as DNA methylation and histone deacetylation, which can gradually suppress gene expression. This epigenetic silencing leads to a time-dependent decrease in therapeutic protein expression levels, further limiting the longevity of the treatment (57).

Recent technical improvements have led to more persistent effects, lasting up to 4–5 months in some studies. These advancements include the development of AAV variants less prone to epigenetic silencing and the use of promoters that resist methylation. However, achieving longer and more stable therapeutic effects remains a challenge, particularly in the cochlea compared to the vestibule, where phenotype correction has shown a wider therapeutic window and longer persistence of effects. The differential response between cochlear and vestibular tissues may be partly due to tissue-specific differences in epigenetic regulation and the cellular environment’s impact on vector performance. This discrepancy is further highlighted by a proof-of-concept study where TMC1 gene replacement intervention on P14 and P30 restored balance but had no positive influence on hearing (23).

It’s worth noting that retinal cells can maintain their function throughout their lifespan after gene therapy transduction, making gene therapy for retinal diseases promising for potentially offering long-lasting vision restoration. Unlike retinal cells, inner ear hair cells undergo functional changes over time, which significantly challenges the long-term efficacy of gene therapy approaches for hearing disorders (58). These findings emphasize the need for targeted approaches that can achieve long-lasting effects specifically in the cochlea, matching the efficacy observed in vestibular treatments.

## Epigenetics of developing hair cells

**Figure 2:**
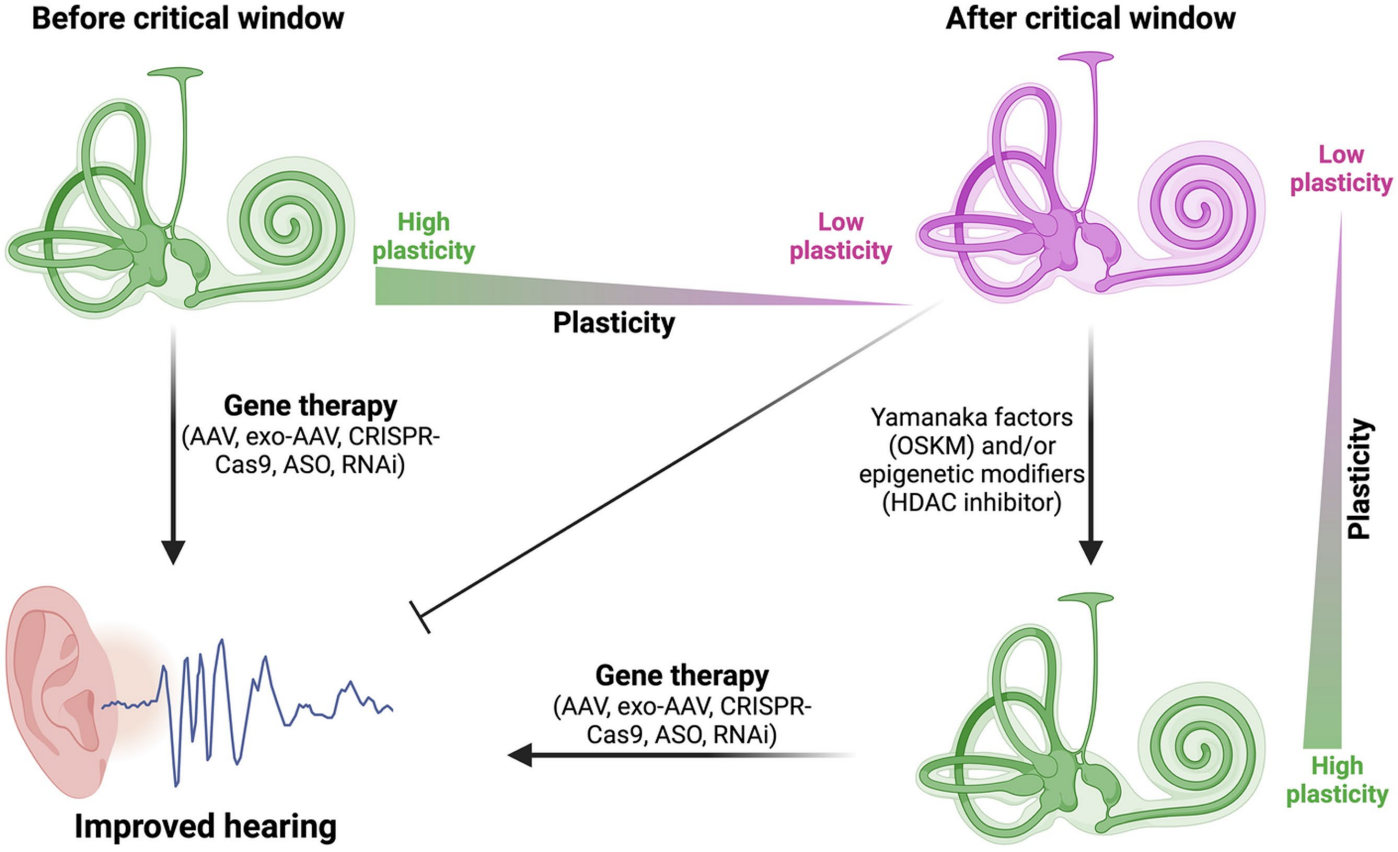
Expanding the critical window for gene therapy in sensorineural hearing loss. This figure illustrates how Yamanaka factors (OSKM) and/or epigenetic modifiers (such as, HDAC inhibitors) can potentially increase plasticity in mature cochlear cells after the critical window. This increased plasticity may allow gene therapy approaches to be effective even in later stages, potentially expanding the treatment opportunities for sensorineural hearing loss.

Epigenetic mechanisms orchestrate gene expression without altering the underlying DNA sequence (59–61). Briefly, these heritable changes primarily occur through two key processes: DNA methylation and histone modifications, with chromatin remodeling and non-coding RNA interactions playing secondary roles. DNA methylation involves the addition of methyl groups to cytosine bases within CpG dinucleotides, typically resulting in gene silencing. This process is executed by DNA methyltransferases (DNMTs), including DNMT1, DNMT3A, and DNMT3B, while demethylation is facilitated by Ten-Eleven Translocation (TET) enzymes. These enzymes collectively play crucial roles in maintaining cellular identity, genomic stability, and embryonic development. Histone modifications encompass a variety of chemical alterations to histone proteins. Histone acetyltransferases (HATs) add acetyl groups to lysine residues, generally promoting gene activation, while histone deacetylases (HDACs) remove these groups, often leading to gene repression. Similarly, histone methyltransferases (HMTs) and demethylases (HDMs) add or remove methyl groups, respectively, with varying effects on gene expression depending on the specific residue modified. These modifications collectively modulate chromatin structure, thereby regulating DNA accessibility and transcriptional activity. One exciting possibility is that a combinatorial approach with HDAC inhibitors and gene therapy may prove more effective than either approach alone (Figure 2) (62–64).

Inner ear development, particularly hair cell formation, is intricately regulated by these epigenetic processes (65–67). During early embryogenesis, the *de novo* methyltransferase DNMT3A plays a crucial role in otic placode formation by modulating the methylation status of key developmental genes such as *GBX2*, *Pax2*, and *Sox10*. As development progresses, the interplay between DNA methylation and histone modifications becomes increasingly significant in defining cellular potential and fate. The transition from progenitor cells to hair cells exemplifies the dynamic nature of epigenetic regulation and cellular plasticity. The histone demethylase LSD1 (KDM1A) is essential for maintaining progenitor cell identity by catalyzing H3K9 demethylation at otic progenitor genes (68, 69). As cells commit to the hair cell lineage, significant epigenetic changes occur, particularly in the regulation of the pro-hair cell gene Atoh1 (70). Initially, the Atoh1 locus exhibits bivalent histone marks, with both activating (H3K4me3) and repressive (H3K27me3) modifications present. During hair cell differentiation, this bivalency resolves: levels of activating H3K4me3 increase while repressive H3K27me3 levels decrease. Concurrently, H3K9 acetylation levels rise, promoting Atoh1 expression. Moreover, the base-to-apex gradient of Atoh1 regulation during cochlear development needs to be considered when designing therapeutic interventions, as this developmental pattern may influence treatment efficacy across different cochlear regions (71). This precise orchestration of epigenetic modifications ensures the appropriate temporal and spatial patterning of hair cell development within the cochlea, gradually establishing the unique transcriptional profile of mature hair cells while progressively restricting cellular plasticity.

## Navigating cellular plasticity: the promise of partial reprogramming

Partial reprogramming has emerged as a promising approach to increase cellular plasticity and epigenetic flexibility without fully reverting cells to a pluripotent state (72–74). It aims to temporarily reset certain epigenetic marks associated with aging, allowing for beneficial rejuvenation effects while avoiding the risks of complete reprogramming to pluripotency. Partial reprogramming involves the transient expression of pluripotency factors like Oct4, Sox2, Klf4, and c-Myc (OSKM) for a limited duration. Ocampo et al. demonstrated that cyclic short-term induction of OSKM in mice could extend lifespan and ameliorate features of aging in multiple tissues without tumor development (75). The timing and dosage of reprogramming factor expression is critical, as Ohnishi et al. found that reprogramming after longer periods can lead to tumor formation (76). Studies in the inner ear highlight the critical role of Myc family genes in regulating cellular plasticity and proliferation. For instance, transient expression of c-Myc in SOX2-expressing otic progenitors creates immortalized multipotent otic progenitor cells capable of differentiating into functional hair cells and neurons (77), while n-Myc has been shown to be essential for proper inner ear development and morphogenesis through its regulation of cell proliferation (78). These findings underscore the importance of precise temporal control of Myc expression in both development and potential regenerative strategies.

Recent studies have shown that partial reprogramming can reset various epigenetic marks associated with aging (79). For instance, Ocampo et al. observed that cyclic OSKM expression in mice restored youthful levels of H3K9me3 and H4K20me3 histone modifications in certain tissues (75). This epigenetic remodeling appears to be a key mechanism by which partial reprogramming increases cellular plasticity and promotes rejuvenation. Mah et al. further demonstrated that even brief exposure to reprogramming factors can induce significant changes in the epigenetic landscape, including alterations in DNA methylation patterns (80). These epigenetic changes may allow cells to temporarily access a more plastic state, potentially enabling them to adopt more youthful characteristics or respond more effectively to their environment. As research in this field progresses, careful optimization of reprogramming protocols will be crucial to maximize the rejuvenation benefits while minimizing risks associated with increased plasticity.

## Epigenetic rejuvenation as a catalyst for improved gene therapy outcomes: a speculative perspective

The integration of epigenetic rejuvenation techniques with gene therapy approaches presents an intriguing avenue for enhancing the efficacy of treatments for sensorineural hearing loss (SNHL). By leveraging partial reprogramming using Yamanaka factors (OSKM) and various epigenetic modifiers, we may be able to increase the plasticity of cochlear cells beyond their normal developmental window. This increased plasticity could potentially make mature hair cells and supporting cells more responsive to gene therapy interventions, even in fully developed auditory systems (Figure 2).

Expanding the critical window for effective intervention is a key challenge in SNHL gene therapy. The use of OSKM factors and epigenetic modifiers could potentially extend this window by temporarily reverting mature cochlear cells to a more youthful, plastic state (Figure 2). This approach could be particularly beneficial for addressing conditions like Cx26 mutation-induced hearing loss, which manifests at birth, by creating a more permissive environment for genetic corrections or replacements in fully developed cochlear cells. More recently, work in mice showed that Eps8 KO mice fail to respond positively to AAV-mediated expression of Eps8 after P2 (81), providing a useful critical window to explore for expansion via partial reprogramming or HDAC inhibitors.

Moreover, epigenetic rejuvenation techniques could potentially enhance the longevity of therapeutic effects. By creating a more stable and receptive cellular environment for the introduced genetic material, these approaches might help maintain the expression of therapeutic genes over a longer period. This could be particularly impactful in addressing the differential response observed between cochlear and vestibular systems, potentially bringing the durability of cochlear treatments closer to the more persistent effects seen in vestibular interventions.

In conclusion, while significant challenges remain, the combination of epigenetic rejuvenation techniques and gene therapy holds great promise for expanding the treatable window and enhancing the longevity of therapeutic effects in SNHL. Future research should focus on optimizing the timing, dosage, and specific combinations of these approaches to maximize their benefits while minimizing potential risks. This innovative strategy could potentially revolutionize our ability to address genetic hearing impairments, offering hope for more effective and durable treatments for individuals across a wider age range and at various stages of hearing loss progression.

## Data Availability

The original contributions presented in the study are included in the article/supplementary material, further inquiries can be directed to the corresponding author.
